# A Method for Detecting Long Non-Coding RNAs with Tiled RNA Expression Microarrays

**DOI:** 10.1371/journal.pone.0099899

**Published:** 2014-06-17

**Authors:** Sigrun Helga Lund, Daniel Fannar Gudbjartsson, Thorunn Rafnar, Asgeir Sigurdsson, Sigurjon Axel Gudjonsson, Julius Gudmundsson, Kari Stefansson, Gunnar Stefansson

**Affiliations:** 1 Faculty of Physical Sciences, University of Iceland, Reykjavik, Iceland; 2 deCODE Genetics, Reykjavik, Iceland; CSIR Institute of Genomics and Integrative Biology, India

## Abstract

Long non-coding ribonucleic acids (lncRNAs) have been proposed as biomarkers in prostate cancer. This paper proposes a selection method which uses data from tiled microarrays to identify relatively long regions of moderate expression independent of the microarray platform and probe design. The method is used to search for candidate long non-coding ribonucleic acids (lncRNAs) at locus 8q24 and is run on three independent experiments which all use samples from prostate cancer patients. The robustness of the method is tested by utilizing repeated copies of tiled probes. The method shows high consistency between experiments that used the same samples, but different probe layout. There also is statistically significant consistency when comparing experiments with different samples. The method selected the long non-coding ribonucleic acid PCNCR1 in all three experiments.

## Introduction

It has been predicted that more than 30,000 non-protein coding genes are associated with the human genome [1], [2]. They can vary considerably in length, as the shortest products, micro RNAs (miRNA), are on average only 22 bp wheras long non-coding RNA (lncRNAs) are at least 200 nucleotides. An excellent overview of lncRNAs is given in Baker et al. [3].

Several publications indicate that lncRNAs might play an important role in cancer development [4]–[10]. Non-coding RNAs have been identified that distinguish between different prostate tissue types and can predict clinical outcomes for primary tumors [11]. LncRNAs are also thought to play a regulatory role in cancer-associated pathways governing mechanisms such as cell growth, invasion, and metastasis and have been seen to be expressed differently in primary and metastatic cancer and to wire up cancer growth [12], [13]. LncRNAs originate mainly in long stretches in the genome where no protein-coding genes have been identified [3]. An example of such a chromosomal region is 8q24.2 [14] where the lncRNA PCNCR1 (AB458446) has been identified [15]. Notably, multiple single nucleotide polymorphisms (SNPs) of 8q24 have been found to associate with increased risk of developing prostate cancer [16]–[19]. LncRNAs might both serve as new targets in cancer therapy, as well as serve as an extensive source of new biomarkers [20]–[23]. Currently, at least 11 databases which record lncRNAs [7], [24]–[27].

Microarrays are one of the most commonly used technologies to locate RNA genes. A typical microarray contains hundreds of thousands of spots and each spot contains multiple copies of the same DNA oligonucleotides, known as probes. The probes on the microarray are hybridized to a labeled RNA sample and the array is subsequently washed. Theoretically this will result in the labeled sample only remaining at the spots where the sample hybridized to probes. The signal intensities at the corresponding location on the microarray are used as a measure of the relative abundance of hybridization of each probe. Tiled microarrays contain probes that overlap and cover a fairly large target part of the genome. They have been successfully used to assess expression of non-coding RNAs and transcription in “gene deserts” [28]–[30].

Gene-expression signals in microarrays are affected by several sources of variation [31], [32]. Further issues and different biases arise when using tiled microarrays, as opposed to other analyses of differential expression [33]. It is therefore important to take technical variation into account when doing statistical analyses on microarray data [33], [34]. Various microarray platforms are available and the importance of testing the same biological samples on different platforms has been stressed [28]. Consistency and repeatability of differential expression in microarray experiments has also been widely studied [35], [36], but less is known about the repeatability of findings in tiled micorarray experiments. Expression levels are generally lower for lncRNA than protein coding genes, [7] and the reliability of detection of low expression genes has been questioned [37], [38].

Recent methods of detecting regions of activity include the use of a wavelet transformation in order to target regions of activity from noisy data, and the TileShuffle method, which has been shown to detect differently expressed segments in tiling arrays with lower false discovery rates under equal sensitivities than commonly used methods [39], [40]. The TileShuffle method has, however, shown a serious lack of repeatability, even with the same samples in the same batch of experiments [42]. This could (partially) be explained by the fact that, (monitored with enough accuracy), expression levels measured by every single probe differ, so searching for “expressed” regions is a somewhat futile exercise [41].

The primary aim of this study is to design a method which detects candidate regions for containing lncRNAs with good consistency. The hypothesis is that locating fairly long regions (approx 1,500 nt) with the highest ratio of probes expressed above the median over the whole region will give more consistent results than searching for shorter regions with the highest expression levels as conventional methods do. This increased consistency will presumably result in reduced sensitivity for detecting expressed areas. The proposed selection method is therefore not targeted at finding all expressed areas, but rather to identify which areas are expressed and to do so in a highly consistent manner.

In the following, the method will be run on three different experiments with Nimblegen microarrays, all of which contain probes tiling a part of the 8q24.2 region. The three experiments have different array design containing samples from prostate cancer patients. Some of the samples vary from experiment to experiment while others are used in repeated experiments. These would ideally assist in locating lncRNA genes.

A secondary aim of the study is to identify regions on the 8q24 region that are candidates for containing the loci of lncRNA genes correlated with prostate cancer development.

## Materials and Methods

### Ethics Statement

The tissue samples used in this study were collected in conjunction with a study on the genetics of prostate cancer. The study was approved by the National Bioethics Committee (approval #00/103) and Data Protection Authority of Iceland (approval #2001020228). All prostate cancer cases in Iceland were invited to participate and written informed consent was obtained from all participants. Personal identifiers were encrypted by a third-party encryption system for which the Data Protection Authority maintains the code.

### Experimental Overview

The data used in this paper were RNA expression data from three different sets of custom designed Nimblegen microarray, exclusively for these three experiments. In each experiment the array contained tiled probes from chr8∶127,640,000–129,120,000 at locus 8q24. In this paper a selection method is proposed which is fine-tuned in Experiment 1 and Experiment 2 and validated in Experiment 3. A summary of the main settings of each experiment is found in Table 1 and the array design and description of the samples for each experiment is detailed below.

**Table 1:** Experimental settings.

Description:	Experiment 1	Experiment 2	Experiment 3
Samples used:	7 independent	7 independent	3 repeated, 3 pairs
Number of arrays:	7	7	9
Number of repeated spots:	none	none	10
Number of containers:	24	none	10
Isothermal probes:	no	yes	no

Overview of the experimental settings for the three experiments.

### Samples

Each of the first two experiments consisted of seven arrays containing the same seven samples, extracted from normal prostate tissue of prostate cancer patients. In the third experiment, one of these seven samples (sample number five) was used repeatedly on three arrays and in addition three pairs of both normal and tumor tissue from three prostate cancer patients were used. The Gleason grading score of the tumors for these seven samples is shown in Table 2 along with the age at diagnosis.

**Table 2:** Tumor grading.

sample	age at diagnosis	Gleason score
array 1	68	8
array 2	66	6
array 3	59	7
array 4	59	6
array 5	55	7
array 6	66	6
array 7	66	6

Gleason grading score of the tumors for the samples used in Experiment 1 and Experiment 2. The sample on array 5 was used repeatedly on three arrays in Experiment 3.

DNA was synthesized from Total RNA (Clonetech) using the High capacity cDNA reverse transcriptase kit (Applied Biosystems Inc) at the deCODE lab for all samples in all experiments. Labeling and hybridization was performed by NimbleGen Systems Inc., Madison, WI USA, following their standard operating protocol.

### Probe-set and Array-layout

In the first experiment, the whole area was tiled with 60 nt probes at a 10 base interval. All probes with blat score greater than 5, [43] or blast score greater than 40, [44] were excluded from the statistical analysis. These probes were excluded a priori from the probe sets in Experiments 2 and 3, but a posteriori for Experiment 1. Excluded probes were 8,723 out of 147,009 or 5.9%. Experiment two contained isothermal probes, which were not evenly spaced over the area, whereas Experiment 3 contained 50 nt probes tiled at a 20 base interval.

Spatial artifacts in the expression signal were minimized in Experiment 1 and 3 by aggregating the wells of the microarray into non-overlapping logical “containers”. Experiment 1 used 24 containers and each set of 24 consecutive tiled probes was allocated to different containers, randomly allocated within each. In Experiment 3 each probe was replicated 10 times, and each replicate was allocated to a different container.

The data have been deposited in NCBI’s Gene Expression Omnibus [46] and are accessible through GEO Series accession number GSE45934 (http://www.ncbi.nlm.nih.gov/geo/query/acc.cgi?acc=GSE45934). The exact probe layout on the arrays in each experiment is listed at the GEO cite in the .ndf files.

### Proposed Selection Method

The primary objective of this study was to develop a statistical method to select a fixed number of smaller regions which consistently show high expression levels in a tiled microarray experiment. Thus the selection method should, with high probability, select the same areas in repeated experiments.

The method proposed is as follows:

The region of interest (8q24) is split up into even-sized regions. The proportion of probes with signal intensities above the median (0) in each region is subsequently calculated and ranked over all probes. The sum of the rank over all the arrays in the corresponding experiment is then calculated and a fixed number of regions with the highest rank sum are selected.

Some regions only contained few probes included in the analysis. Therefore only regions containing at least one probe for each 25 base interval on average are considered eligible for selection.

### Normalization and Monte-carlo Simulation

In order to avoid spurious correlations, the only normalization applied was to take the logarithm of the data, subtract the median within each container and divide by the median absolute deviation (MAD). In the case of no containers (Experiment 2), the median and MAD over the whole array were used.

The third experiment used 10 repeated spots for each probe, evenly spread across the array. This permitted Monte-Carlo simulation of the expression signals, assisting in minimizing some of the biases caused by technical variation. The Monte-Carlo simulations also assist in estimating the robustness of the method and sensitivity to the number of underlying samples used.

All statistical analyses were performed in the R statistical package and graphics were generated with the ggplot2 library [47], [48].

## Results

### Determining the Optimal Region Length and Proportion of Regions Selected

The proposed selection method requires both the length of each region and the number of regions selected to be determined. In order to estimate the optimal region length and the number of regions to be selected, the method was run for the first two experiments with these parameters varying and the agreement between experiments investigated. Those experiments were run independently, with different array design, but the same set of seven samples was used in both experiments. The difference in array design can result in some regions being eligible in one of the experiment but not the other. It was therefore ensured that regions were considered eligible if and only if they were eligible by the above criteria in both experiments.

**Figure 1:**
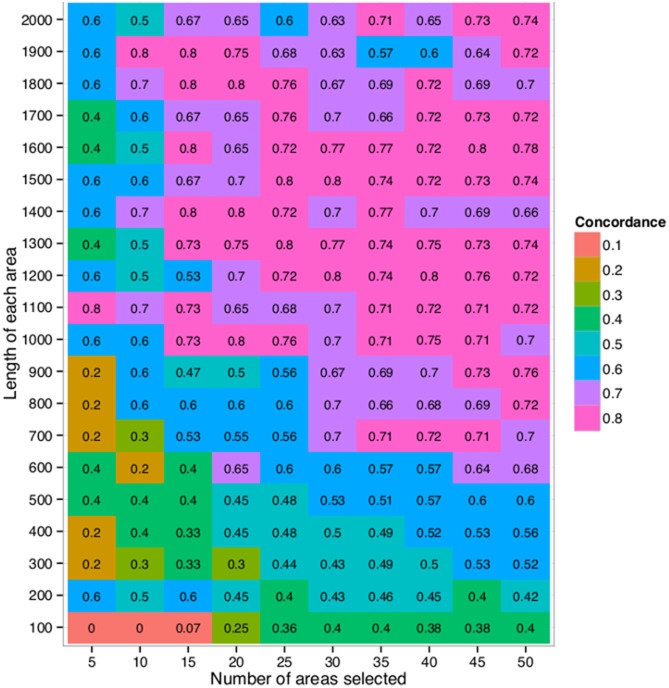
The proportion of regions that were selected in both Experiment 1 and Experiment 2. The underlying region was split up into equally sized regions and a fixed number of regions with the highest ratio of probes, within the region, expressed above the median, was selected. The proportion of regions that were selected in both Experiment 1 and Experiment 2 was calculated for varying length of each underlying region (y-axis) and the total number of regions to be selected (x-axis). The numbers within each cell show the exact proportions for the corresponding criteria.

Figure 1 shows the agreement between the two experiments. The number of regions selected ran from 5, 10, 15 and up to 50 regions, and their length varied from 100, 200, 300 and up to 2,000 bases. Selecting the 25 highest ranked regions (top 3%) of length 1,300 bases was one of the options that gave the most concordance between the two experiments, where 20 (80%) of the 25 regions selected in the first experiment were also selected in the second experiment. That corresponds to choosing the top 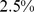 of expressed regions. The location of the first bp of the 1,300 bp according to both hg18 and hg19 of the twenty regions that were chosen in the first two experiments are listed in Table 3, along with a brief description of the genes reported at these loci.

**Table 3:** Regions selected in first two experiments.

Hg18 location	Hg19 location	genes
127,717,200	127,648,018	TCONS_00015165
128,094,200	128,025,018	PCAT1
128,167,000	128,097,818	PRNCR1
128,194,300	128,125,118	nothing yet
128,251,500	128,182,318	TCONS_00015169
128,338,600	128,269,418	nothing yet
128,819,600	128,750,418	MYC exon
128,876,800	128,807,618	MYC exon
128,882,000	128,812,818	MYC and Pvt1 introns
128,898,900	128,829,718	MYC and Pvt1 introns
128,911,900	128,842,718	MYC and Pvt1 introns
128,987,300	128,918,118	MYC and Pvt1 introns
128,989,900	128,920,718	Pvt1 intron
128,991,200	128,922,018	Pvt1 intron
129,008,100	128,938,918	Pvt1 intron
129,023,700	128,954,518	Pvt1 intron
129,025,000	128,955,818	Pvt1 intron
129,026,300	128,957,118	Pvt1 intron
129,027,600	128,958,418	TMEM75
129,028,900	128,959,718	TMEM75

The loci of the 20 regions selected in first and second experiments, according to hg18 and hg19 and the genes reported at these loci.

There were in total 1,006 regions of length 1,300 bases. The probability of selecting 20 or more of the regions twice when choosing randomly the 25 out of 1,006 regions with the maximum expression is
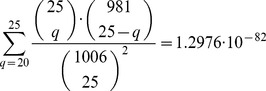
(1)


The concordance was tested for in total 200 combinations, so with a Bonferroni correction, the multiplicity-adjusted P-value becomes 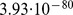, still highly significant.

### Agreement between Independent Experiments

Having chosen a selection method for finding regions of elevated expression levels, based on the first two experiments, this method was run again on the dataset obtained from the third experiment. That experiment also contained probes from 8q24 and samples from prostate cancer patients, but the samples were not the same as in experiments 1 and 2. The same parameters as obtained from the analysis of the first two experiments were used, i.e. 25 regions of length 1,300 bases with the highest rank sum of the average expression levels over the whole region, were selected. In this experiment, each probe was repeated 10 times on the array. Therefore the median expression level of every 10 identical probes was used as the expression signal at the corresponding location. Since the repetitions are distributed across the containers, this automatically corrects for any spatial trends across the array.

**Figure 2:**
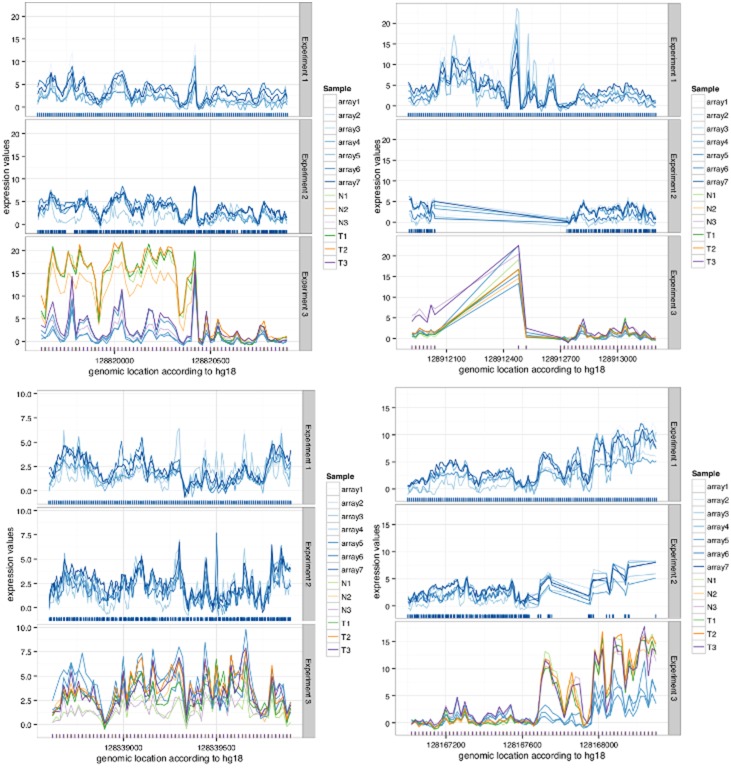
The signal intensities on the regions that were selected in all three experiments. On x-axis is the genomic location of the probes on chromosome 8q24. On y-axis are the signal intensities of the probe at the corresponding location. One line is drawn for each array where the colouring represents the sample used on the array. These are drawn separately for the results from Experiment 1 (top), Experiment 2 (middle) and Experiment 3 (bottom). The tick-marks on the x-axis denote the locus of the probes at 8q24.

All of the regions eligible according to the previously described criteria were eligible in the third experiment, such that the set of 1,006 underlying regions remained the same as before. This time, four of the 20 regions that were selected in the first two experiments were reselected in the third experiment and five of the 25 regions that were selected in either of the first two experiments were also selected in the third experiment. The normalized RNA expression levels of the four selected regions are shown on Figure 2. The location of the first bp of the 1,300 bp fragment according to hg18 is depicted above the corresponding graph.

The probability of selecting 4 or more of the same 20 regions when choosing randomly 25 out of 1,006 is
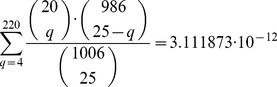
(2)


which also is statistically significant.

In the following, the top 25 regions selected in each experiment will be called the *experiment-wise selected regions*.

### Robustness Estimated by Monte Carlo Simulations

In Experiment 3, each probe is repeated 10 times. By randomly selecting which one of those ten replications represent the signal at every location a Monte Carlo simulation is produced to form pseudo-replications of each region. The robustness of the selection method was tested by creating 10,000 such simulations of the tiled regions and applying the region-selection method on each simulation.

**Figure 3:**
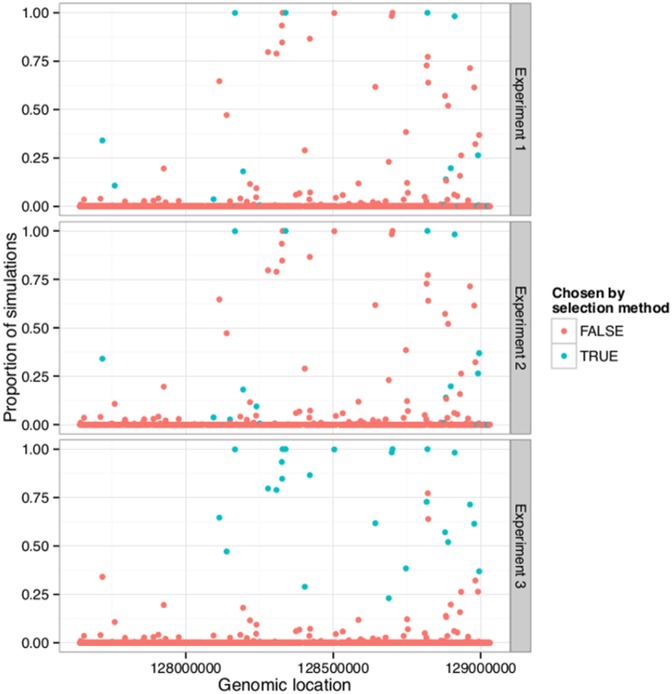
The number of Monte Carlo simulations for which each region is chosen by the selection method using arrays with different samples. The genomic location of the regions on 8q24 is on the x-axis. The proportion of Monte Carlo simulations for which the region was chosen is on the y-axis. The graph is shown with two different colourings, representing whether the region was among the previously experiment-wise selected regions (cyan) or not (pink). Those who were selected previously in Experiment 1 are shown at the top graph, Experiment 2 in the middle and Experiment 3 at the bottom. The simulations are done on the ten repeated spots for each probe for all nine arrays in Experiment 3.

Figure 3 shows a graph of the proportion of Monte Carlo simulations for which each region was chosen among the top 25. The colouring indicates whether the region was among the 25 experiment-wise selected regions for Experiments 1, 2 and 3. It is seen that the majority of regions are never among the top 25, whereas 14 regions are selected in at least 75% of the simulations. The experiment-wise selected regions seem to be selected more often in the Monte-Carlo simulation.

This hypothesis can be tested with Wilcoxon rank sum tests of the null hypothesis of equality of median frequency of selection in the Monte-Carlo simulation of these two groups of regions (those selected in a particular experiment compared to those who were not selected). The statistics for each experiment become 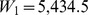, 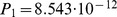, 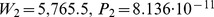 and 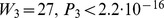. The null was thus rejected in all three cases.

### Robustness with the Same Sample on Fewer Arrays

The first two experiments contained the same seven samples, whereas Experiment 3 contained seven samples of which one was also used in experiments 1 and 2, but the other six were from three pairs of normal vs tumor tissue. See Table 1. The sample also used in the first two experiments was placed on three arrays in Experiment 3.

**Figure 4:**
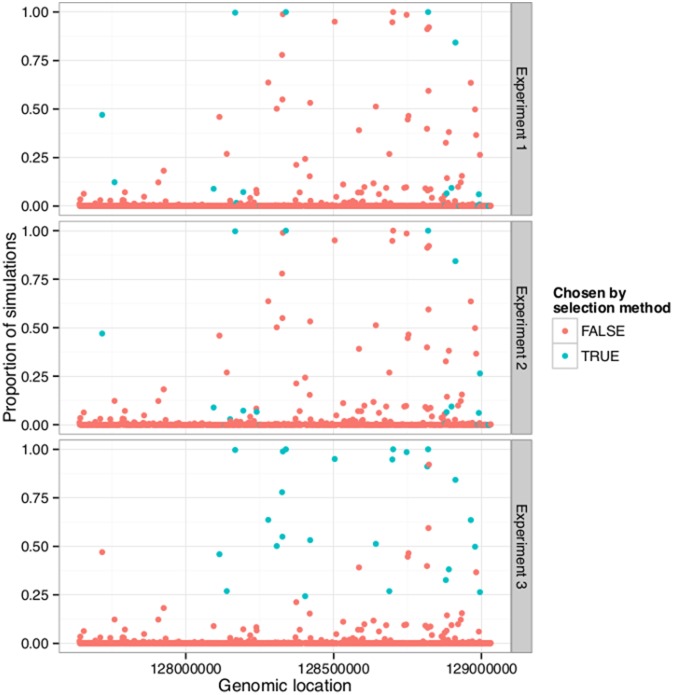
The number of Monte Carlo simulations for which each region is chosen by the selection method using arrays with the same sample. The genomic location of the regions on 8q24 is on the x-axis. The proportion of Monte Carlo simulations for which the region was chosen is on the y-axis. The graph is shown with two different colourings, representing whether the region was among the previously experiment-wise selected regions (cyan) or not (pink). Those who were selected previously in Experiment 1 are shown at the top graph, Experiment 2 in the middle and Experiment 3 at the bottom. The simulations are done on the ten repeated spots for each probe for the three arrays in Experiment 3 that contained the same sample.

An obvious question is whether the selection method would show more agreement between the three experiments if it was only applied to the sample that was used in all three experiments. Thus the Monte-Carlo simulation was run again but now with only the three arrays that contained the same sample. Figure 4 shows the same type of figure as shown in Figure 3, but now the colouring represents the results from applying the selection method and the Monte-Carlo simulation to only the repeated sample.

Now the concordance is less than the one obtained by using all available samples. Fewer regions are never selected and 12 regions are selected in at least 75% of simulations. The hypothesis of whether the experimental-wise selected regions were selected more often in the Monte Carlo simulations was tested as before. The statistics are: for Experiment 1 were W = 5,795, p-value = 7.193 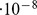, for Experiment 2, W = 6,043.5, p-value = 2.227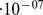, and for Experiment 3, W = 611.5, p-value 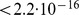. Thus, the null hypothesis for the corresponding Wilcoxon rank sum tests was rejected again in all three cases.

### Further Details on the Four Selected Regions

The location of the first bp of the 1,300 bp according to both hg18 and hg19 of the four regions that were chosen in all three experiments are listed in Table 4, along with a brief description of the gene reported at these loci. The regions correspond to the oncogene Myc known to be over-expressed in prostate cancer [49], [50] along with the oncogene Pvt1 [51], a Myc protein target which is over-expressed in transformed cells. The third region is at the location of PRNCR1 a known lncRNA also associated with prostate cancer [15]. Nothing has been reported yet at the fourth location.

**Table 4:** Regions selected in all three experiments.

hg18 location	hg19 location	genes
128,819,600	128,750,418	MYC exon
128,911,900	128,842,718	Pvt1
128,167,000	128,097,818	PRNCR1
128,338,600	128,269,418	nothing yet

The loci of the selected regions according to hg18 and hg19 and the genes reported at these loci.

## Discussion

In this paper a method for detecting elevated expression levels for regions (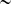
1,000 kb) of moderate RNA expression was introduced. It is demonstrated how this can be used to locate lncRNAs in humans (or other species). The method splits the region of interest into equally sized regions, calculates the proportion of probes with signal intensities above the median within each region and selects the 2.5% of regions with highest rank sum over arrays.

This method is fairly easy to implement and is independent of various experimental specifics of the array layout and probe design and the microarray platform used. It is therefore applicable e.g. for metadata analyses of microarray data from different platforms.

The method was applied to two independent microarray experiments, which had different array designs, but the same set of samples. The method was set to select the 25 regions with the highest average expression levels for each experiment. This choice is based on guaranteeing consistency in the selection. This resulted in 20 regions being selected in both experiments. When compared with a third experiment, where different samples were used, 4 out of the regions selected in both of the first two experiments were selected again. The analysis indicates that the different array design has a small effect on the selection method.

The number of samples in all experiments considered here is small. In general one would expect a larger sample size (i.e. more than seven biological samples in experiments 1 and 2) to lead to a more consistent selection of regions, as this is the usual effect of increased sample size, at least when a fixed number of regions is selected as is the case here.

The third experiment contained 10 repeated copies of each probe on each array. Monte Carlo simulations of the signal intensities of the regions were undertaken in order to estimate the robustness of the selection method.

It is important to note that the method is more robust when applied to all nine arrays in Experiment 3 than when only applied to the three arrays containing the same sample. Although a larger experiment is needed for verification, this would seem to imply that a larger sample size implies greater consistency.

Sample preparation was done according to the same protocols on the same labs for all three experiments so the variation due to these factors should be minimal. This indicates that the signals detected are prevalent in different subjects and also that there is considerable variability in the signals from array to array, even though the same sample is being used.

Four regions were consistently chosen in all three experiments. Three of them correspond to genes associated with prostate cancer, but no reports have been made on the fourth location. It could thus be a candidate locus for a lncRNA, possibly correlated with prostate cancer.

RNA sequencing is a rising alternative to tiled microarrays that provides improved accuracy in several regards and has been used successfully to discover novel non-coding RNAs [45]. However, the development of methods for targeting ncRNAs with tiled microarrays still remains of importance for at least two reasons. First, tiled microarrays are still less expensive than RNA-sequencing, although the cost of RNA-sequencing is continuously decreasing. Second, much data from tiled microarray experiments exist which can be further utilized with better statistical methods. Finally, confirmation of novel findings still needs to be done, e.g. by real-time PCR.

The proposed selection method may be useful as an add-on to conventional data analysis pipelines to further identify the most concordant and significantly expressed transcripts once basic data analysis has been performed.

## Conclusion

The proposed method locates regions with elevated expression levels in RNA expression microarrays with good consistency. It is particularly promising as an add-on to conventional data analysis and succeeds in locating regions containing known lncRNAs on locus 8q24 and proposes a candidate region where no lncRNAs have been reported.
